# Hearing in Noise Test, HINT-Brazil, in normal-hearing children^☆^

**DOI:** 10.1016/j.bjorl.2017.04.006

**Published:** 2017-05-09

**Authors:** Carolina Lino Novelli, Nádia Giulian de Carvalho, Maria Francisca Colella-Santos

**Affiliations:** Universidade Estadual de Campinas (UNICAMP), Faculdade de Ciências Médicas, Centro de Investigação em Pediatria, Campinas, SP, Brazil

**Keywords:** Speech, Hearing, Noise, Child, Fala, Audição, Ruído, Criança

## Abstract

**Introduction:**

The auditory processing is related to certain skills such as speech recognition in noise. The HINT-Brazil test allows the measurement of the Speech/Noise ratio however there are no studies in the national literature that establish parameters for the child population.

**Objective:**

To analyze the performance of normal-hearing subjects aged 8–10 years old in tasks for speech recognition in noise using HINT test.

**Methods:**

Sixty schoolchildren were evaluated. They were between 8 and 10 years of age, of both genders, and had no auditory and school complaints, with results ranking within normality for the Basic Audiological Assessment and the Dichotic Digits Test. HINT-Brazil test was applied with headphones, with the Speech/Noise ratio in conditions of frontal noise, noise to the right, and noise to the left being investigated. The software calculated the Composite Noise, which corresponds to the weighted mean of the tested conditions.

**Results:**

There was no statistically significant difference between the ears, nor between the genders. There was a statistically significant difference for age ranges of 8 and 10 years, in situations with noise, and for Composite Noise. The age group of 10 years showed better performance than the age group of 8; the age group of 9 years did not show statistically significant difference regarding the other age ranges. We suggest the values of mean and standard deviation of the Speech/Noise ratio, considering the age ranges of: 8 years – Frontal Noise: −2.09 (±1.09); Right Noise: −7.64 (±1.72); Left Noise: −7.53 (±2.80); Composite Noise: −4.86 (±1.31); 9 years – Frontal Noise: −2.82 (±0.74); Right Noise: −8.49 (±2.24); Left Noise: −8.41 (±1.75); Composite Noise: −5.63 (±1.02); 10 years – Frontal Noise: −3.01 (±0.95); Right Noise: −9.47 (±1.43); Left Noise: −9.16 (±1.65); Composite Noise: −6.16 (±0.91).

**Conclusion:**

HINT-Brazil test is a simple and fast test, and is not difficult to performed with normal-hearing children. The results confirm that it is an efficient test to be used with the age range evaluated.

## Resumo

**Introdução:**

O processamento auditivo está relacionado a determinadas habilidades, como o reconhecimento de fala no ruído. O teste HINT-Brasil permite a mensuração da relação Fala/Ruído, porém, não há na literatura nacional estudos que estabeleçam parâmetros para a população infantil.

**Objetivo:**

Analisar o desempenho de normo-ouvintes, de 8 a 10 anos, nas tarefas de reconhecimento de fala no ruído, por meio do teste HINT.

**Método:**

Avaliados 60 escolares, entre 8 e 10 anos, de ambos os gêneros, sem queixas auditivas e escolares, e com resultados dentro da normalidade para a Avaliação Audiológica Básica e o Teste Dicótico de Dígitos. O teste HINT-Brasil foi aplicado com fones de ouvido, sendo pesquisada a relação Fala/Ruído nas condições Ruído Frontal, Ruído à Direita e Ruído à Esquerda. O software calculou o Ruído Composto, que corresponde à média ponderada das condições testadas.

**Resultados:**

Não houve diferença estatisticamente significante entre as orelhas, nem entre os gêneros. Houve diferença estatisticamente significante para as faixas etárias de 8 e 10 anos, nas situações com ruído e para o Ruído Composto. A faixa etária de 10 anos apresentou desempenho melhor do que a faixa etária de 8; a faixa etária de 9 anos não apresentou diferença estatisticamente significante com relação as outras faixas etárias. Sugerimos os valores de Média e Desvio Padrão da relação F/R, considerando as faixas etárias: 8 anos - RF: -2,09 (±1,09); RD: -7,64 (±1,72); RE: -7,53 (±2,80); RC: -4,86 (±1,31); 9 anos - RF: -2,82 (±0,74); RD: -8,49 (±2,24); RE: -8,41 (±1,75); RC: -5,63 (±1,02); 10 anos - RF: -3,01 (±0,95); RD: -9,47 (±1,43); RE: -9,16 (±1,65); RC: -6,16 (±0,91).

**Conclusão:**

O teste HINT-Brasil é um teste simples e rápido, e não oferece dificuldades em seu uso com crianças normo-ouvintes; os resultados mostram se tratar de um teste eficaz para ser utilizado com a faixa etária avaliada.

## Introduction

Communication enables humans to create and transform the environment where they live, being decisive in their quality of life. Communication is mainly expressed through oral language, which requires an intact auditory system, so that the individual can listen, understand and process knowledge, resulting in learning.

The auditory system consists of the peripheral and central parts. The peripheral part is that whose main function is to capture and transmit the sound wave to the cochlea, where it will be processed and sent to the central part through the vestibulocochlear nerve. The central auditory system is responsible for the functions of sound discrimination, location, recognition, comprehension, selective attention and auditory memory. Thus, the complexity of the central auditory nervous system allows the analysis of sound events, from the simplest to the most complex messages, such as speech.^1^

Hearing loss is defined as a deviation or a change for the worse in the auditory structure or function.^2^ The central auditory processing refers to the series of processes involved in the auditory functions that were already described, referring to the mechanisms that are basically performed by the structures of the central auditory nervous system.^3^ We can therefore say that the auditory processing disorder can be defined as a deficit in at least one of these mechanisms.

Since the 1950s, researchers have studied children who presented audiograms within normal range, but with auditory complaints. The symptoms described indicated good auditory acuity in acoustically controlled places, but hearing difficulties in real listening environments. Thus, the existence of a deficit in the auditory perception was found, mainly regarding the suppression of a competitive noise to pay attention to another.^4^

The perception of speech has always been of great interest to those who work with human communication. To evaluate and diagnose how impaired this perception is, several tests are used in clinical practice, but most of them use isolated stimuli with mono and dissyllable words.^5^, ^6^

Jacob et al.^7^ highlighted the importance of conducting tests in the presence of noise, because the results of evaluations of patients with the same speech recognition abilities in silence may be completely different in competitive situations of speech. Thus, the tests used in the Logoaudiometry, which is part of the Basic Audiological Assessment, are not as effective in detecting how the functional capacity of the individual perceives and understands speech in noisy environments, because they are applied in silence. The evaluation of the auditory ability of children in these conditions is even more complex.

A child with speech comprehension difficulties in the presence of noise may be unable to use his/her language knowledge to improve the degraded signal. On the other hand, his/her difficulty in recording the properties of the acoustic signal may be so deficient that even the full use of language knowledge could not lead to satisfactory understanding; thus, the problem results in an inability to process auditory signals.^8^

Therefore, the inclusion of Hearing in Noise Tests (HINT) in the auditory evaluation of schoolchildren becomes essential because these tests more closely resemble the speech situations of the individuals, and ensure that the evaluation is performed in a real environment of daily listening.

For better evaluation of speech recognition in the presence of competitive stimulus, the use of sentences is superior than the use of words, because the sentences simulate the situations of daily communication better.^9^

It is therefore important that research is carried out in the area of speech perception and central auditory processing, with types of noise that are representative of the daily listening situation, in order to evaluate its effectiveness in clinical practice.^10^

The HINT is an adaptive test for the measurement of the Sentence Recognition Threshold (SeRT), which proves the statistical and practical efficiency of hearing in the noise through sentences of everyday life. It can be used to assess auditory functional capacity, i.e., to determine how well the person is able to hear and understand in noisy environments. It consists of digitally recorded sentences, which can be presented in silence and noise; the masking noise used in the HINT test is a white noise, which was synthesized at the original sample rate and scaled to the same amplitude of the sentences.^11^ The sentences were standardized for language, difficulty, intelligibility and phonetic distribution.

The HINT method was developed by the House Ear Institute (HEI) in 1994, being initially tested in normal-hearing adults, so that parameters could be obtained for other groups.^11^

For the development and application of HINT in Brazil, a material of controlled sentences was initially elaborated. The material was elaborated in a work of partnership between researchers of the Universidade Estadual de Campinas and Universidade de São Paulo, in Bauru, which standardized the test with a native Brazilian speaker.^12^

A Brazilian study with adults, when comparing HINT with the speech perception tests applied in clinical audiology, observed that HINT allowed measuring difficulties in normal-hearing subjects.^13^

We found in the national literature 11 studies that used HINT as a method to evaluate speech recognition in noise. Of these, four were performed with the child population, but only one included children with hearing within normal limits in the sample, with 21 children between 7 and 14 years old.^7^ In addition, SeRT research was conducted in free field, with no use of headphones.

HINT test has, in the international literature, some adapted versions for children, which consist of a subset of the lists of sentences of the original HINT, but they are recorded in the silence, by children. These studies indicated that the HINT version for this age group can be used clinically in the assessment of speech recognition in noise in individuals with different hearing disorders, and also with those with normal hearing.^14^, ^15^, ^16^, ^17^

Although some studies have applied HINT-Brazil in the child population,^5^, ^7^, ^18^, ^19^, ^20^ because the speech material developed seeks to control the variables that can influence speech intelligibility for adults and children,^7^ the software does not provide a test version for this population, as in other languages. Thus, it is fundamental that there are instruments containing more complex tasks to detect the difficulties of speech perception in noise of school age children, since the learning disorders can be associated with difficulties of auditory processing in the classroom.

Therefore, it is necessary to elaborate evaluation protocols that aim to analyze the performance of children with no hearing impairment in linguistic tasks in the presence of noise, so that the normality thresholds can be established and used in the evaluation of children with both peripheral and central hearing disorders.

Thus, the objective of this study is to evaluate the performance of normal-hearing children between the ages of 8 and 10 in the tasks of speech recognition, in silent settings and in noise, using HINT test, considering the age group and the female and male genders.

## Methods

This work is a descriptive, observational, cross-sectional, prospective study developed at the Institution where the research was carried out. It was approved by the Ethics Committee under no. 785723.

A total of 60 schoolchildren were evaluated, of which 32 were female and 28 were male, with ages ranging from 8 to 10 years, from Elementary School of the Municipal School Network.

The subjects were divided into three groups: 22 children aged 8 years in Group I, 18 children aged 9 years in Group II, and 20 children aged 10 years in Group III. Data collection was carried out from August 2014 to January 2016.

Initially the children were selected through a questionnaire answered by the pedagogical team of the selected schools. The questionnaire consisted of questions regarding the school performance of each student, their attention and behavior, and the presence of evidence of hearing difficulties in the child. The questionnaires were analyzed, and only the children with adequate school performance, good behavior, who were attentive and had no evidence of auditory alterations were selected.

Next, a telephone contact was made with the parents or guardians of each child, for explaining the procedures that would be performed, the voluntary nature of the research, the objectives of the study, and the absence of health risk. Those who agreed were invited to go to the place of data collection with the child. On the scheduled date, the person responsible for the child signed the Free and Informed Consent Form.

Inclusion criteria were: age range of 8–10 years, to be a student in the public school system, not to have difficulties at school, not to have complaints and/or hearing problems, and to have normal results in the Basic Audiological Assessment and the Dichotic Digit Test.

As exclusion criteria, the following was considered: to have difficulties at school, to have speech and hearing complaints, to have a history of otitis, to be on the use of psychoactive drugs, to present hearing loss or unilateral or bilateral alterations of the auditory structure or function and to have mental, neurological, and/or genetic syndromes.

The following procedures were performed:

Anamnesis: carried out with parents so that children with hearing loss and/or a history of recurrent otitis media, children who had already undergone speech therapy, and possible other complaints such as inattention and difficulties in oral language acquisition, reading and writing were excluded from the study.
Meatoscopy: allowed to observe the presence of obstacles to the performance of the test;
Basic Audiological Evaluation: consisting of the tests: pure tone audiometry, Logoaudiometry, Immittanciometry.
Dichotic Digits Test: The Free Attention stage of this test was used as a procedure for screening the auditory processing, as it allows for evaluation of the figure-background ability for verbal sounds through binaural integration.^21^
HINT-Brazil test: Children who had results within the parameters of normality for the performed procedures were referred for the HINT test, in the situation with headphones.

The HINT test kit contains the Hearing Test Device (HTD) microprocessor, version 7.2, manufactured in 2003, by Bio-Logic System Corp. and developed by HEI's Laboratory of Hearing Devices Research of the Science and Human Communication Department. This equipment contains a software that conducts the test process with the sentences recorded, and the concurrent noise of the white noise type. TDH 39 headphones were used.

HINT consists of digitally recorded sentences, which can be presented in silence or noise, with headphones or speakers, in a free field. Each HINT-Brazil test list has 20 sentences and the test administration time varies from 8 to 10 min.

Sentences are presented by a male speaker, in silence and noise; as suggested by Bevilacqua,^12^ in the study that performed the standardization of the Brazilian version of HINT, the noise is fixed at 65 dB (A), and the speech signal intensity varies according to the repetitions, that is, at each correct answer the intensity of the speech decreases, and at each error the intensity increases.

The initial speech intensity is 65 dB (A), i.e., Signal to Noise Ratio (S/N = 0); during the presentation of the first four sentences, variations of 4 in 4 dB (A) occur, which allows the estimation of the subject's threshold. From the fifth sentence, the variation becomes 2 by 2 dB (A), and the threshold of each test condition is determined, after the presentation of the 20 sentences of the selected list.^22^

Four lists were selected, out of 12 contained in the HINT-Brazil material. The order of presentation of the lists was fixed for all children.

The software allowed the determination of the SeRT, in dB, in the condition: Speech in Silence (SS): It also allowed the determination of the lowest Speech/Noise (S/N) ratio in which the subject correctly repeated 50% of the sentences presented, in the following conditions: frontal noise (FN); noise to the right (RN); noise to the left (LN).

**Figure 1:**
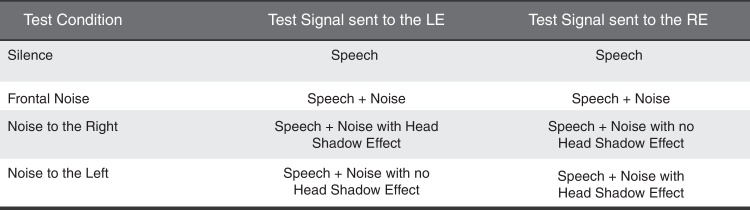
Test conditions with the use of headphones.

Each of these test conditions corresponds to a particular situation of silence or noise as indicated in Fig. 1.

The tests performed with headphones are presented with a digital filtering, which simulates the conditions and characteristics related to the position of the head that would occur in the tests carried out in free field.^23^

At the beginning of the test, the students were asked to repeat the sentences they heard, even if incomplete or incorrect. The sentence should be precisely repeated, to be considered correct; however, small substitutions in verbs and articles, for example, were allowed, as directed by Nilsson.^11^

The strategy used allowed the determination of the S/N ratio in each test condition. At the end, the software computed the Composite Noise (CN), which is the weighted mean of the S/N ratio in the three noise conditions, from the formula CN = (2 × FN + RN + LN)/4.

Data were tabulated, and the following statistical analyzes were carried out: comparison between genders; comparison between ages; comparison between the right and left ears.

Statistical analysis of the collected data was performed using the software “The SAS System for Windows”, version 9.4. A significance level of 0.05 (5%) was adopted and indicated by an asterisk (*) in the results. Mann–Whitney test was used to compare the results between genders, and the Kruskal–Wallis test was used to compare the results between ages; the comparison of the results between right/left ears was through Variance Analysis (ANOVA) for repeated measures.

## Results

We present the results obtained in the HINT-Brazil test, regarding SeRT, under the conditions tested, comparing the values obtained in relation to the variables gender and age and ears. Table 1 shows the characterization of the sample, according to the three age groups and male and female genders. There was no statistically significant difference for the sample number considering the male and female genders.

**Table 1:** Sample characterization, according to the age group and gender.

	8 years	9 years	10 years	Total	*p*-Value
Male	9	9	10	28	0.5552^a^
Female	13	9	10	32	
Total	22	18	20	60	

a Kruskal–Wallis test.

Table 2 shows the values of mean and SD of the S/N ratio, of the male and female genders, regardless of age, in the test conditions of SN, FN, RN and LN, and CN. The results do not indicate a statistically significant difference in relation to this variable.

**Table 2:** Male and female children, according to the performance on SS, FN, RN and LN test conditions and in CN.

S/N ratio	Female			Male			*p*-Value
	*n*	Mean	SD	*n*	Mean	SD	
SS	32	22.07	3.18	28	23.00	2.86	0.2859^a^
FN	32	−2.74	0.83	28	−2.47	1.20	0.3425^a^
RN	32	−8.80	1.64	28	−8.17	2.21	0.1519^b^
LN	32	−8.73	1.93	28	−7.89	2.51	
CN	32	−5.75	1.00	28	−5.27	1.40	0.1380^b^

*n*, sample number; SD, standard deviation; SS, Speech in Silence; FN, frontal noise; RN, noise to the right; LN, noise to the left; CN, composite noise.

a Mann–Whitney test.

b ANOVA comparison for repeated measures.

Table 3 shows the mean and SD values of the S/N ratio, ages 8, 9 and 10 years, regardless of gender, in the test conditions SN, FN, RN, and LN, and CN. The results point to a statistically significant difference for the noise conditions, and for CN calculation, regarding the comparison between the ages of 8 and 10 years.

**Table 3:** Children in the 8-, 9- and 10-year age group, according to their performance in the SS, FN, RN and LN test conditions, and CN.

S/N ratio	8 years			9 years			10 years			*p*-Value
	*n*	Mean	SD	*n*	Mean	SD	*n*	Mean	SD	
SS	22	22.97	2.82	18	23.09	3.32	20	21.46	2.90	0.2332^a^
FN	22	−2.09	1.09	18	−2.82	0.74	20	−3.01	0.95	0.0078 (8 > 10)^a^
RN	22	−7.64	1.72	18	−8.49	2.24	20	−9.47	1.43	0.0048 (8 > 10)^b^
LN	22	−7.53	2.80	18	−8.41	1.75	20	−9.16	1.65	
CN	22	−4.86	1.31	18	−5.63	1.02	20	−6.16	0.91	0.0035 (8 > 10)^a^

*n*, sample number; SD, standard deviation; SS, Speech in Silence; FN, frontal noise; RN, noise to the right; LN, noise to the left; CN, composite noise.

a Kruskal–Wallis test.

b ANOVA comparison for repeated measures.

**Figure 2:**
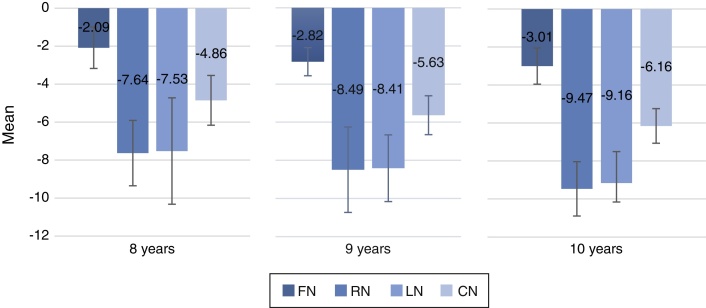
Children of 8-, 9- and 10-years age groups, according to their performance in the SS, FN, RN and LN test conditions, and CN.

Fig. 2 shows the mean and SD values of the S/N ratio, ages 8, 9 and 10 years, regardless of gender, in noise and CN conditions. Lower values of the S/N ratio indicate better performance.

Table 4 presents the general descriptive analysis of the study, with the values of mean, median, standard deviation (SD), S/N ratio minimum and maximum, for each test condition and for the calculated CN, regardless of gender and age. There was no statistically significant difference between the ears in the comparison between the RN and LN test conditions.

**Table 4:** Mean, median, standard deviation, minimum and maximum for the SS, FN, RN and LN test conditions, and CN.

S/N ratio	*n*	Mean	Median	SD	Minimum	Maximum
SS	60	22.5	22.4	3.05	15.10	30.50
FN	60	−2.61	−2.75	1.02	−4.8	0.80
RN	60	−8.51	−8.80	1.94	−11.90	−2.10
LN	60	−8.34	−8.40	2.24	−12.80	1.20
CN	60	−5.53	−5.70	1.22	−7.60	−1.40

*p*-Value RN × LN = 0.2670 (ANOVA comparison for repeated measures).

SeRT, Sentence Recognition Threshold; *n*, sample number; SD, standard deviation; SS, Speech in Silence; FN, frontal noise; RN, noise to the right; LN, noise to the left; CN, composite noise.

## Discussion

HINT is a test that aims to analyze the auditory closure ability, by means of sentences, and therefore it was chosen to be the instrument of evaluation of this study. It was performed in an acoustically treated environment with headphones. The use of headphones allows a virtual sound field to be created, where the hearing of both ears can be evaluated simultaneously and separately; in addition, it prevents errors in the results due to the evaluated person's head involuntary movements.^11^

The sample consisted of 60 individuals, 32 females and 28 males, thus being homogeneous in terms of gender (Table 1).

When analyzing the results with respect to the female and male gender, there was no statistically significant difference in any of the test situations or in the CN (Table 2).

The Brazilian study that contained normal-hearing children in the sample did not present information regarding gender,^7^ therefore it could not analyze if there was a difference for this variable. Sbompato et al., in a study with a population of adults, and that aimed at the standardization of HINT-Brazil for this population, as well as the present research, did not find significant differences as to gender.^24^

The studies by Jerger,^25^ Garcia^26^ and collaborators analyzed the speech recognition in the noise through the test of sentence recognition with competitive message called PSI; again, both studies did not find significant differences between genders.

Thus, despite the scarcity of studies in the national literature with the same characteristics of this research, the studies that were found confirm the absence of interference of the gender variable in the responses of individuals in speech recognition tasks in noise.

Regarding the age group, the sample was divided into three groups, of 8, 9 and 10 years old, with 22 individuals in the 8-year age group, 18 in the 9-year age group, and 20 in the 10-year age group. The mean age was 8.97 years (±0.84).

We consider it important to analyze this age group since it is the crucial age for the development of school skills, and because their hearing abilities are already assessable. According to American Speech-Language-Hearing Association (ASHA), due to the great variability in the results, it is recommended that the tests used for auditory processing are not administered in children under 7 years of age.^27^

When comparing the results regarding age group, in the SS test condition (Table 3) the differences presented between the groups were not statistically significant. However, considering the comparison of the results regarding the age group in the test conditions of FN, RN and LN, as well as in CN, the difference presented was statistically significant when comparing the age groups of 8 and 10 years, with the 8-year-old group having an average of S/N ratio higher than the 10-year group individuals, i.e., they had a worse performance (Table 3).

A Canadian study of the application of HINT in French, with a sample of 70 children, found a variation in the S/N ratio in the condition with noise among the age groups, with the decrease of SeRT being statistically significant according to age increase.^14^

These findings are in agreement with those obtained in a study that evaluated, in children aged 8–10 years, with and without school difficulties, the processing of certain auditory abilities, among them speech recognition in noise. The study obtained the improvement of the answers with the increase of age, with this improvement being statistically significant for the age group of 10 years, in comparison with the age group of 8 years; the authors considered the age group of 9 as a transition age, resembling either the 8-year age group or the 10-year age group.^25^

Myhrum et al. stated that the children population has more limited vocabulary, and less knowledge of language rules compared to adults; thus, children tend to present greater difficulty in understanding language signs, especially when these are sentences.^15^

Boyd and Bee^28^ stated that the reticular formation, the encephalic region in charge of the attentional processes, has its myelination started during the first 2 years of life, but this process continues throughout childhood and adolescence, and is completed only around 20 years. Since the processes of choosing the important sound message to the detriment of another are regulated by attention, it can be stated that the performance in the Closure task is gradually improved with the increase of age, and that the ability of speech recognition in the noise is based in the functioning of auditory pathways that depend on maturation of the pathways.^1^

According to studies performed at the *HEI*, for HINT speech materials in American English, every 1 dB increase in SeRT corresponds to about 10% poorer intelligibility in noisy listening conditions.^23^

Thus, we then suggest the values of mean and SD of the S/N ratio, for the noise test conditions and the CN, considering the ages of 8, 9 and 10 years (Table 3).

Finally, Table 4 shows the values for the general mean, regardless of age or gender, in all test situations.

Regarding the FN test condition, the total mean S/N ratio was -2.61. This value is higher when compared to −6.5 found in an American study about HINT-Brazil in young adults^29^ and also to the value of −4.6 found in the Brazilian Portuguese HINT,^12^ a standardization study of HINT also in adults. These studies are in line with the findings by Vaillancourt et al.,^14^ whose analysis demonstrated that SeRT reaches adult values only in older children. Regarding the findings of a Norwegian study with children, however, mean values between −0.3 and −3.3 were obtained in the FN condition,^15^ coinciding with our findings.

Regarding CN, this was obtained by calculating CN = (2 × FN + RN + LN)/4. This calculation equalizes the contribution of the FN and the two lateral tests (RN and LN), thus providing a single general index of speech recognition in noisy environments.^23^

CN overall mean, obtained in the present study, was −5.53 (Table 4). Again, comparing with the findings of the Norwegian study, values between −5.7 and −9.7 were obtained, that is, Norwegian children had better performance under the conditions evaluated.^15^ These variations may be related to the difference between the age groups of the two studies, since the international research was carried out with children between 6 and 13 years old, thus integrating children up to 3 years older than those in the current research; in addition, the aforementioned study was performed in a free field, that is, in different listening conditions.

Considering the conditions in which auditory closure ability was evaluated as a dichotic task, in RN and LN test situations general mean values of −8.51 and −8.34 were obtained, respectively, in our study; however, there is no statistically significant difference between the ears (Table 4). Similarly, another study also did not observe a difference between the ears when applying HINT-Brazil in normal-hearing children in a free field.^7^

A recent study that sought to establish standards of normality for young adults in HINT-Brazil obtained values of −12.1 and −12.2 for RN and LN, respectively, values that are lower than those obtained in this study, because the sample had subjects in the age range between 18 and 50 years.^12^ This fact is in agreement with the statement by Myhrum et al. that the values obtained for speech recognition in noise in children are further away from values obtained in adults, especially in tasks that require lateralization.^15^

Finally, this study may prove that HINT-Brazil test is a simple and rapid test, and does not pose difficulties for its use with normal-hearing children with no complaints at school; the children were attentive and willing to perform well in the test, which made HINT an attractive and easy to apply instrument. In addition, the results that do not indicate a statistically significant difference between the genders or between the ears show that it is an effective test to be used with the age group evaluated.

Therefore, it is important that new research evaluating the closure ability, using the HINT test in children, is performed, so that the results can be compared.

## Conclusion

Based on the results of this research, in which HINT test was applied to 60 normal-hearing schoolchildren aged 8, 9 and 10 years, the following conclusions were obtained:

- No statistically significant differences were observed regarding HINT results and the variables side of the ear and female and male gender;
- The 8-year age group presented a statistically significant difference compared to the 10-year age group, with the latter showing better performance than the 8-year age group, for both noise and CN situations; the 9-year age group did not show a statistically significant difference compared to the 8- and 10-year age groups, in any test condition, nor for CN;
- We suggest the values of mean and SD of the S/N ratio obtained, considering the age groups: 8 years – FN: −2.09 (±1.09); RN: −7.64 (±1.72); LN: −7.53 (±2.80); RN: −4.86 (±1.31); 9 years – FN: −2.82 (±0.74); RN: −8.49 (±2.24); LN: −8.41 (±1.75); CN: −5.63 (±1.02); 10 years – FN: −3.01 (±0.95); RN: −9.47 (±1.43); LN: −9.16 (±1.65); CN: −6.16 (±0.91).

## Conflicts of interest

The authors declare no conflicts of interest.
